# Digital Signal Processing by Virtual Instrumentation of a MEMS Magnetic Field Sensor for Biomedical Applications

**DOI:** 10.3390/s131115068

**Published:** 2013-11-05

**Authors:** Raúl Juárez-Aguirre, Saúl M. Domínguez-Nicolás, Elías Manjarrez, Jesús A. Tapia, Eduard Figueras, Héctor Vázquez-Leal, Luz A. Aguilera-Cortés, Agustín L. Herrera-May

**Affiliations:** 1 Micro and Nanotechnology Research Center, Universidad Veracruzana, Calzada Ruiz Cortines 455, Boca del Río 94294, Veracruz, Mexico; E-Mails: rauljuarez@uv.mx (R.J.-A.); saudominguez@uv.mx (S.M.D.-N.); 2 Depto. Control Automático, Centro de Investigación y de Estudios Avanzados del IPN (CINVESTAV-IPN), Av. IPN 2508, Col. Zacatenco 07360, D.F., Mexico; 3 Institute of Physiology, Benemérita Universidad Autónoma de Puebla, 14 Sur 6301, Colonia San Manuel, Puebla 72570, Puebla, Mexico; E-Mails: elias.manjarrez@correo.buap.mx (E.M.); xchojesus@gmail.com (J.A.T.); 4 Microelectronic Institute of Barcelona IMB-CNM, CSIC, Bellaterra 08193, Spain; E-Mail: eduard.figueras@imb-cnm.csic.es; 5 Electronic Instrumentation and Atmospheric Sciences School, Universidad Veracruzana, Gonzalo Aguirre Beltran S/N, Xalapa 91000, Veracruz, Mexico; E-Mail: hvazquez@uv.mx; 6 Depto. Ingeniería Mecánica, DICIS, Universidad de Guanajuato/Carretera Salamanca-Valle de Santiago km 3.5+1.8 km, Salamanca 36885, Guanajuato, Mexico; E-Mail: aguilera@ugto.mx

**Keywords:** digital signal processing, magnetic field sensor, magnetogram, MEMS, silicon resonator, virtual instrument

## Abstract

We present a signal processing system with virtual instrumentation of a MEMS sensor to detect magnetic flux density for biomedical applications. This system consists of a magnetic field sensor, electronic components implemented on a printed circuit board (PCB), a data acquisition (DAQ) card, and a virtual instrument. It allows the development of a semi-portable prototype with the capacity to filter small electromagnetic interference signals through digital signal processing. The virtual instrument includes an algorithm to implement different configurations of infinite impulse response (IIR) filters. The PCB contains a precision instrumentation amplifier, a demodulator, a low-pass filter (LPF) and a buffer with operational amplifier. The proposed prototype is used for real-time non-invasive monitoring of magnetic flux density in the thoracic cage of rats. The response of the rat respiratory magnetogram displays a similar behavior as the rat electromyogram (EMG).

## 1. Introduction

For biomedical applications, superconducting quantum interference devices (SQUIDs) have been used for noninvasive anatomical and functional medical diagnostics involving imaging, and magnetic marker monitoring of disintegrating and non-disintegrating tablets, capsules and pellets in the gastrointestinal tract [1–4]. The SQUIDs are the most sensitive magnetic field sensors currently available. They operate at low temperature based on two effects: flux quantization and Josephson effects, thus these sensors need a sophisticated infrastructure that increases their size and cost. Giant magnetoresistive (GMR) and Hall sensors have been used for medical diagnosis and bioscreening through electrical detection and biological labeling of superparamagnetic particles (magnetic beads) [5–7]. In addition, GMR sensors have been used in hyperthermia therapy for cancer treatment. These sensors can estimate magnetic fluid weight density inside large tumors [8,9]. However, GMR sensors have temperature dependence and offset, and can be damaged by magnetic flux density close to 1 T [10].

On the other hand, magnetic field sensors based on magnetoelectric (ME) composites could be used for biomagnetic measurements in the picotesla regime [11–15]. Recently, several research groups have studied the performance of these sensors [11–21]. They use piezoelectric and magnetostritive laminate composites and could be candidates for noninvasive medical imaging like magneto-encephalography (MEG) or -cardiography (MCG) [11,13,14]. These sensors have advantages of low cost, and high sensitivity and high spatial resolution [11,14,15]. Their resolution can be increased with further improvements in sensor design, vacuum encapsulation, and the use of Microelectro-mechanical Systems (MEMS) or Nanoelectromechanical Systems (NEMS) technologies [11,14–16]. Atomic magnetic field sensors are also near-room-temperature devices with suitable sensitivity for biomagnetic applications such as MEG and MCG [17–21]. They measure magnetic flux density by establishing an average electron spin polarization in an atomic vapour and sensing the resulting flux-dependent shifts in optical properties. These sensors can achieve sub-femtotesla (sub-fT) sensitivities and could be a promising non-cryogenic, low-cost candidate to replace SQUID sensors. Nevertheless, atomic magnetic field sensors have a very narrow dynamic range and generally must operate under shielded environments [17–21]. Recently, atomic magnetic field sensors based on chip-scale microfabrication (e.g., MEMS technology) were the basis of small and low-cost sensors [22–27] that with their flexible optical and electrical wiring, could be located very close to the skull or thorax to measure MCG or MEG signals [22,26].

In addition, MEMS technology has allowed the development of novel sensors [28]. These sensors can have important characteristics such as small size, lightweight, low power consumption, high resolution, and low cost using batch fabrication [29]. Several researchers [30–41] have developed interesting magnetic field sensors based on MEMS technology. However, most of these sensors have only been tested in the laboratory and have not reached commercial use. A large part of this problem is due to the lack of portable systems for signal processing of the magnetic field sensors. Thus, these sensors need signal conditioning systems to process their responses into suitable signals that can be used in data acquisition systems. These systems could then be adapted for potential biomedical applications and magnetic field sensors could thus compete commercially with several conventional magnetic field sensors.

In this paper, we present a semi-portable prototype for real-time non-invasive detection of magnetic flux density of the thoracic cage of anesthetized and ventilated rats. This prototype consists of a MEMS sensor, a signal conditioning system and a virtual instrument. The signal conditioning system contains a precision instrumentation amplifier, a demodulator, a low-pass filter (LPF) and a buffer with operational amplifier. The virtual instrument for digital signal processing includes an algorithm to implement infinite impulse response (IIR) filters, which are developed in Delphi Borland 7.net. This prototype can be used for monitoring magnetic flux density close to nanotesla in some biomedical applications with resolution in the nanotesla range. However, more experimental and theoretical studies to significantly increase the sensitivity and resolution of this sensor type are needed such as an optimal design of the resonant structure, a vacuum packaging, and reduction of the electronic noise.

Following the introduction, the paper is organized as follows: Section 2 describes the MEMS design, signal conditioning system, and the virtual instrument. Section 3 includes the experimental setup and results of a biomedical application using the semi-portable prototype of the MEMS sensor. The paper ends with a conclusion of our work.

## 2. Prototype Design

This section includes the description of the MEMS sensor design and signal conditioning system, as well as its virtual instrument.

### 2.1. MEMS Design

**Figure 1.**
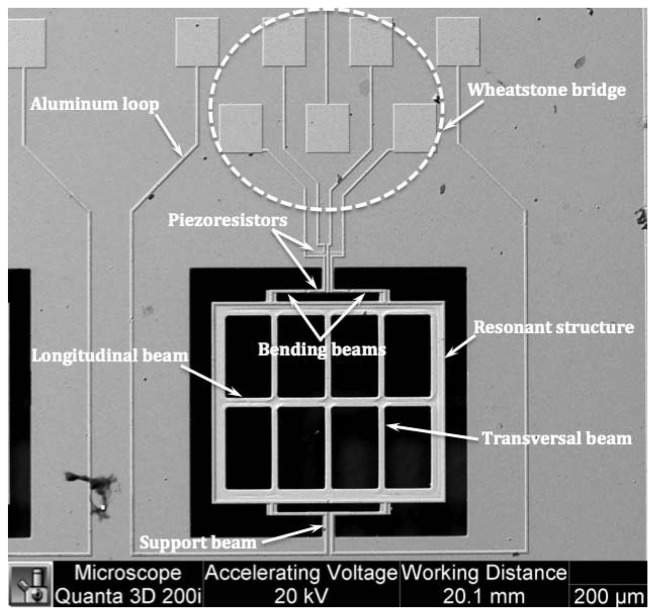
SEM image of a MEMS magnetic field sensor.

The proposed prototype has a MEMS sensor to detect magnetic flux density using the Lorentz force, as shown in Figure 1. This sensor was designed and fabricated by the MEMS group from the Micro and Nanotechnology Research Center (MICRONA) of the Veracruzana University (Veracruz, Mexico) with collaboration of the Microelectronics Institute of Barcelona (IMB-CNM, CSIC, Bellatera, Spain) [38,40]. This sensor has a 700 × 600 × 5 μm resonant structure, formed by a rectangular loop, four bending silicon beams and an arrangement of transversal and longitudinal silicon beams. The resonant structure is joined to a silicon substrate through two torsional beams (60 × 40 × 5 μm). In addition, the MEMS sensor contains a Wheatstone bridge with four p-type piezoresistors, in where two piezoresistors are positioned on two bending beams and others two piezoresistors are located on the surface of the silicon substrate.

**Figure 2.**
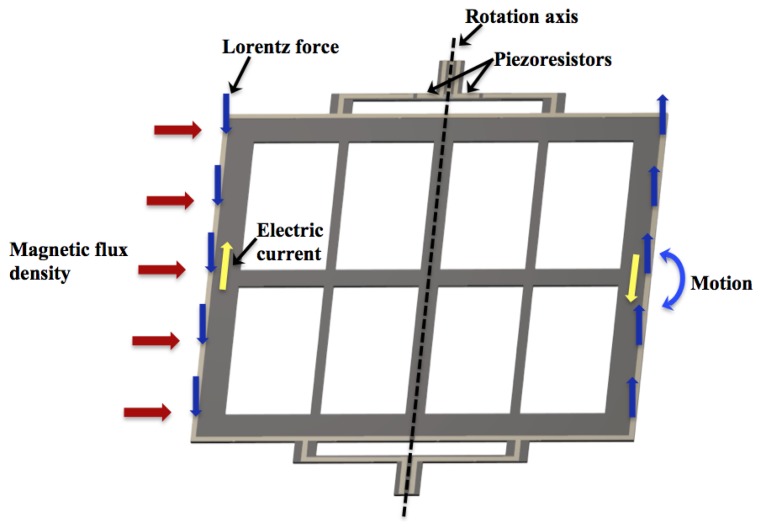
Operation principle of a MEMS magnetic field sensor.

The MEMS sensor operates with the Lorentz force, which is generated by the interaction of a magnetic flux density and a sinusoidal excitation current through an aluminium loop, as shown in Figure 2. This magnetic flux density is applied in the longitudinal direction of the resonant structure. The Lorentz force is amplified when the resonant structure operates at its first resonant frequency. It causes a longitudinal strain in the two piezoresistors located on the bending beams, which changes their initial resistances. It generates a variation in the output voltage of the Wheatstone bridge. Thus, this electrical signal is related with the magnetic flux density applied to the MEMS sensor.

### 2.2. Signal Conditioning System

This section presents the block diagram of the signal conditioning system implemented in a printed circuit board (PCB) for a MEMS magnetic field sensor. It is packaged using a DIP-8 (eight-pin dual in line package). A sensor with similar characteristics was reported in elsewhere [38], which presented an experimental sensitivity and resolution of 4 V·T^−1^ and 1 μT, respectively.

**Figure 3.**
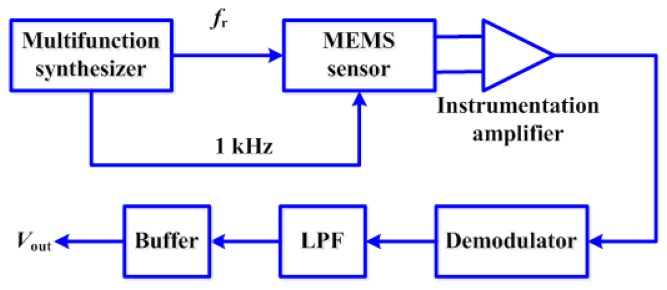
Signal conditioning stages of the MEMS magnetic field sensor.

Figure 3 shows the signal conditioning system in a PCB of our MEMS sensor. It has an instrumentation amplifier, a demodulator, a LPF, and a buffer with operational amplifier. Furthermore, an Agilent 8904A multifunction synthesizer (Agilent Technologies^^®^^, Santa Clara, CA, USA) is used to supply the ac signals to the sensor with two frequencies. A frequency corresponds to the resonant frequency (*f_r_*) of the MEMS sensor, which is used as frequency of the excitation sinusoidal current of the sensor. Another frequency (*f_c_*) of 1 kHz is used to bias the Wheatstone bridge of the MEMS sensor. Thus, an amplitude-modulated (AM) signal (without amplification) of output voltage of Wheatstone bridge in time domain is obtained as:

(1) $$V_{\text{bridge}}\;(t)=\frac{\alpha }{2}\text{cos}(\omega_{c}t)\;\text{s}in\;(\omega_{r}t)$$

where *ω_r_* = 2π *f_r_*, *ω_c_* = 2π *f_c_* and *α* is a parameter proportional to the resistance change of piezoresistors.

An instrumentation amplifier AD524AD (Analog Devices^^®^^, Boston, MA, USA) increases 1,000 times the AM signal of the MEMS sensor. This amplifier generates a noise voltage of 7 nV·Hz^−1/2^ [42]. The ac phase and amplitude information of the AM signal is recovered as a dc signal at the output of the demodulator AD630KN (Analog Devices^®^). This information is related with the polarity and magnitude of the magnetic flux density applied to the MEMS sensor. This polarity is detected connecting the excitation sinusoidal current of the sensor to the phase shifter of the demodulator. A third-order passive LPF, implemented with RC circuits, filters the amplitude information as dc signal, which is proportional to the magnitude of the applied magnetic flux density. This third-order LPF has the following transfer function (*TF_LPF_*):

(2) $${TF}_{\text{LPF}}=\frac{39.75\;x\;10^{4}}{s^{3}+367.64s^{2}+32.44\;x\;10^{3}s+39.75\;x\;10^{4}}$$

The transfer function (*TF_LPF_*) is obtained by the relation between the output voltage of the demodulator and the input of the buffer with an OPA177GP operational amplifier (Burr-Brown Corporation^®^, Tucson, AZ, USA), whose linearity range depends of the passive components values used in the third-order LPF. This buffer is employed for the impedance coupling between the LPF output voltage and the analog input of the PCI-DAS6031 DAQ card (Measurement Computing^®^, Norton, MA, USA). This DAQ card has 16 bits resolution, which improves the stability of the voltages values supplied by the PCB output.

**Figure 4.**
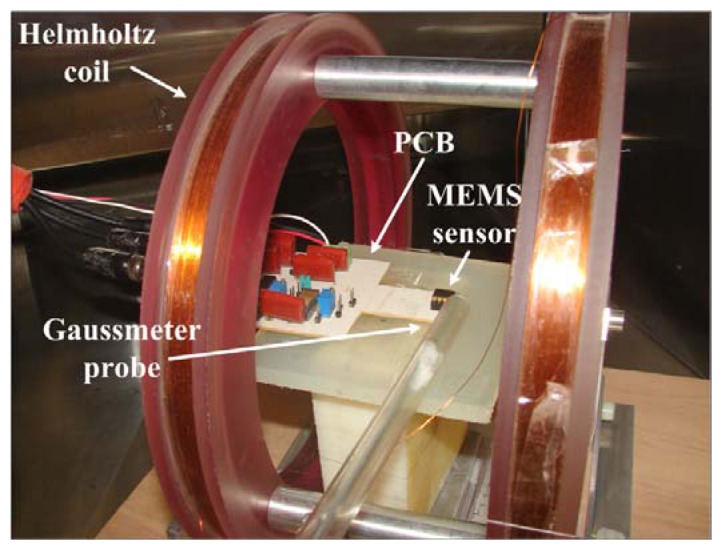
PCB of the MEMS sensor inside a Helmholtz coil.

The signal conditioning system implemented in the PCB (see Figure 4) without virtual instrumentation presents a resolution of 1 μT. However, the resolution of this system can be improved on the order of nanoteslas through a digital signal processing at the PCB output. This signal processing uses a virtual instrument to reduce the noise sources.

### 2.3. Virtual Instrument

This section presents the digital signal processing at the PCB output for the MEMS sensor, which is digitally processed through the infinite-impulse-response (IIR) filters designed as a virtual instrument.

The virtual instrument for MEMS sensor signal processing was developed in Delphi Borland 7.net, which uses object-oriented programming techniques to implement algorithms. This virtual instrument has advantages such as integrity, compatibility, portability, and scalability, which allow the real-time detection of magnetic flux density around of nanotesla. The virtual instrument can control multiple devices (up to 16 input devices and two output devices, both with resolution of 16 bits). In addition, it allows the measurement of MEMS sensor output voltage close to tens of microvolts.

**Figure 5.**
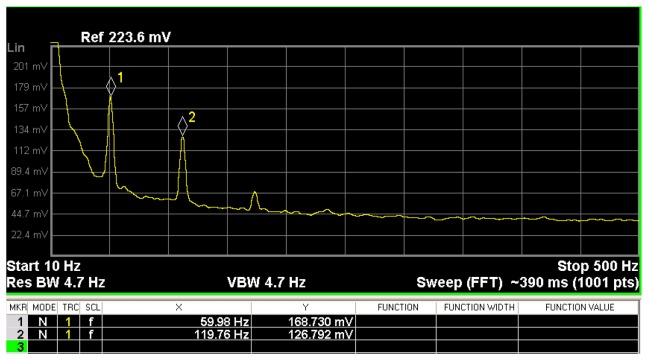
Frequency domain spectrum of the non-filtered PCB output voltage of the MEMS sensor.

In order to find the frequencies in which the electronic noise of the PCB output has the maximum values, it is necessary to measure its frequency domain spectrum. For this, a N9020A spectrum analyzer (Agilent Technologies^®^) is used to evaluate the behavior of the PCB output voltage at the frequency domain considering also its higher harmonics, as shown in Figure 5. By using the Fourier series at the PCB output, the frequency spectrum is represented by:

(3) $$x(t)=\frac{A}{\pi }+\frac{A}{4}\cos(\omega_{o}t)+\sum_{n=2}^{\infty }\frac{2A}{\pi }\frac{{\left(-1\right)}^{\frac{n}{2}+1}}{n^{2}+1}\cos(n\omega_{o}t)$$

where *ω*_o_ is the fundamental frequency and *A* is the magnitude of harmonic. Thus:

$\frac{A}{\pi }=C_{o}^{x}$ represents the fundamental signal of the frequency spectrum.
$\frac{A}{\pi }=C_{1}^{x}$ represents the first harmonic of the frequency spectrum.
$\frac{2A}{\pi }\frac{{\left(-1\right)}^{\frac{n}{2}+1}}{n^{2}+1}\cos(n\omega_{o}t)=C_{n}^{x}$ represents the *n* harmonic of the frequency spectrum.

The signal spectrum of the PCB output is decreased by the factor (1/*a*), in which *a* is the attenuation factor of higher harmonics to control the ripple in the PCB output. Thus, for each frequency between 2 and 345 Hz, where the noise signal spectrum magnitude ${C_{1}}^{x}$ is less than the average noise spectrum magnitude, the output is set to zero. To achieve an output signal approximately equal to zero, a fourth order Bessel band-stop filter can be implemented. By using the Fourier transform in Equation (3), the ${C_{1}}^{y}$ term can be obtained as:

(4) $$C_{1}^{y}=\frac{A}{4}H\;(jW_{o})$$

where ${C_{1}}^{y}$ is the first harmonic of frequency spectrum of the PCB output, *H*(*jW_0_*) is the gain of band-stop filter, and *A*/4 is the first harmonic of frequency spectrum of the PCB output.

Therefore:

(5) $$C_{1}^{y}=\frac{1}{2a}\frac{A}{\pi }$$

The attenuation factor *a* is determined by:

(6) $$a=\frac{2{\left(RC\right)}^{2}\;\omega^{2}}{\pi (1+\omega )}$$

where *ω* is the frequency spectrum of the system, *RC* are the time constant of cut-off frequency obtained through of the analog Bessel filter.

Next, Equation (6) is implemented in the virtual instrument, which contains methods for the communication with the digital I/O of a data acquisition card. It allows the monitoring of the PCB output voltage of the MEMS sensor based on the four-order Bessel band-stop filter. This filter has a gain of 10 and minimum cut-off frequency of 2 Hz and maximum of 345 Hz. It improves the resolution of the PCB output voltage of the MEMS sensor. The Bessel filter is widely used in the industry to implement passive and active analog filters [43]. This filter can increase the flat time and decrease the group delay ripple performance of the filtered signal.

The implemented algorithm in the virtual instrument for the IIR filters considers the configuration of the PCI-DAS6031 DAQ card, function filter settings and the procedures to select source signal and filtered output voltage. Two variables (VFiltered and V) are used to store both filtered and non-filtered PCB output voltages values, respectively. Through the function CbAIn, the analog channel 0 of DAQ card is used as source signal, in which the PCB output voltages values are assigned to the variable V of the virtual instrument. Optionally, this procedure can provide source signals (e.g., sinusoidal, triangular and square waveforms) to simulate the performance of the selected filters in the frequency range from 0 to 60 kHz and voltages values from −4 to 4 Vpp.

On the other hand, a function dspIIRfilter.Filter(V) includes the parameters of IIR filters such as cutoff frequencies, orders and topologies. The filter topologies considered in our virtual instrument are the Butterworth, Bessel and Chevyshev ones. These topologies include the following features: minimum cutoff frequency from 0 to 100 Hz and maximum cutoff frequency from 0 to 500 Hz, filters up to eighth-order and response types of low-pass, high-pass, band-pass, and band-stop. For this research work, we adjust the parameters of the virtual instrument to a fourth-order Bessel band-stop filter and a minimum cutoff frequency of 3 Hz and a maximum cutoff frequency of 345 Hz. The Bessel filter is chosen with these features to filter low-frequency noise and to obtain accurate measurements (nanotesla range) in the semi-portable prototype of the MEMS sensor.

The filter parameters are assigned to the V variable using the function dspIIRFilter.Filter(V) through of the procedure “filtered output voltage”. The time can be defined by the user; thus, the time range (in the virtual instrument) of the filtered and non-filtered PCB output voltage signals can be modified. The minimum and maximum values of time range are 1 ms and 5 ms, respectively. The sampling time of the non-filtered output voltage signal is adjusted to 1 ms for each measured voltage. Next, this voltage is stored in a V variable of the virtual instrument.

The channel 0 of DAQ card is selected as analog output to send in real-time the filtered output voltage signals (VFiltered) multiply by amplification factor. Both filtered and non-filtered output voltage signals are plotted in the virtual instrument using two functions in Delphi Borland 7.net. In addition, these signals can be recorded in a personal computer and exported from the main window of the virtual instrument to a conventional spreadsheet.

**Figure 6.**
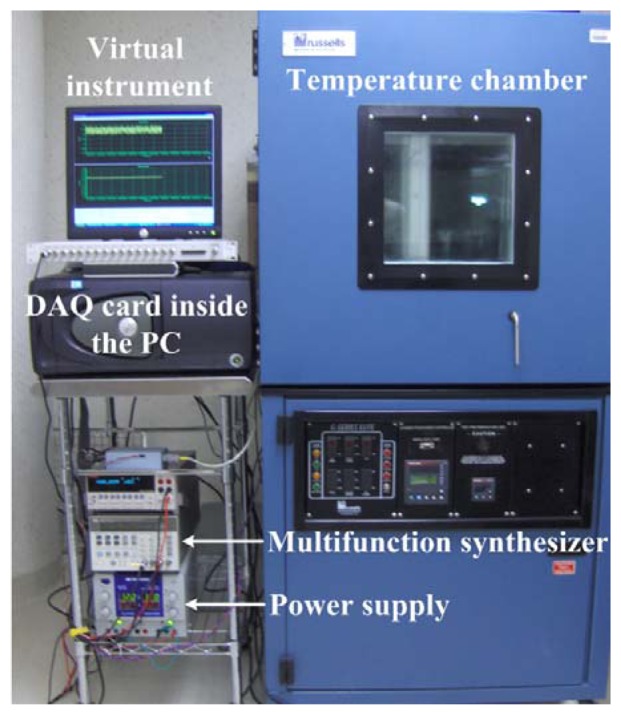
Experimental setup of the MEMS sensor signal processing. The PCB of the sensor is located inside the temperature chamber.

The Figure 6 shows the experimental setup to obtain the MEMS sensor signal processing. This experimental setup includes the virtual instrument developed through Delphi Borland 7.net, as well as a PCB, a Helmholtz coil, a PCI-DAS6031 DAQ card, an Agilent 8904A multifunction synthesizer and a triple output dc power supply (Model 1672 BK Precision^®^, Yorba Linda, CA, USA). This power supply is used to supply the Helmholtz coil and the integrated circuits (IC) of the PCB. The Helmholtz coil is used to generate magnetic flux density, which is applied to the MEMS sensor. For this, the PCB of the MEMS sensor is located inside a temperature chamber (Russells Technical Products^®^, Holland, MI, USA). This chamber is used to keep a constant temperature (25 °C) during the measurements of the prototype output signal in voltage mode. This experimental setup is implemented to obtain the response of the semi-portable prototype output signal (voltage mode) with respect to the applied magnetic flux density.

**Figure 7.**
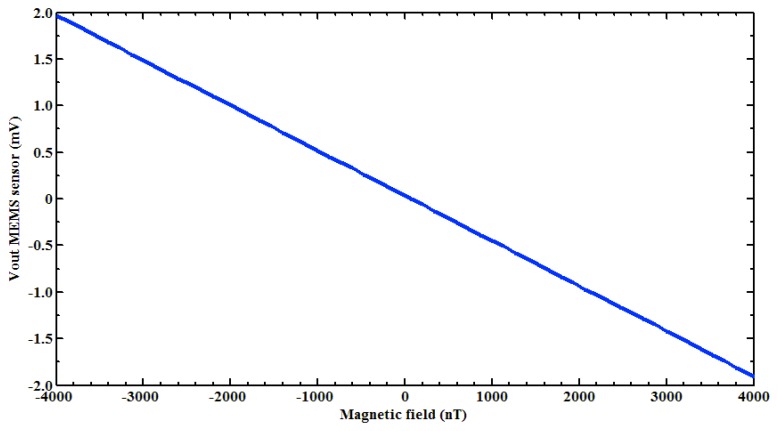
Filtered PCB output voltage (amplified 1,000 times) of the MEMS sensor as a function of the magnetic field density.

Next, a test of the filtered PCB output voltage of the MEMS sensor was made using the digital signal processing. For this, first a Helmholtz coil (Figure 4) was characterized to generate the magnetic flux density in nanotesla range. This coil was supplied with a direct current (dc) voltage range from −1 to +1 V with increments of 10 mV and the magnetic flux density was measured using a digital signal processing of a 475 DSP Gaussmeter (LakeShore^®^, Westerville, OH, USA). Next, the MEMS sensor was placed at the center of the Helmholtz coil and the filtered PCB output voltage was obtained using the designed virtual instrument. This PCB output voltage has an offset of 27 mV (amplified 1,000 times), which was subtracted to only obtain the increment of the PCB output voltage of the MEMS sensor caused by a magnetic flux density (see Figure 7). With our digital signal processing, the electronic noise of the non-filtered PCB output voltage was significantly decreased. It allowed the detection of magnetic flux density on the nanotesla range from −4,000 to +4,000 nT.

**Figure 8.**
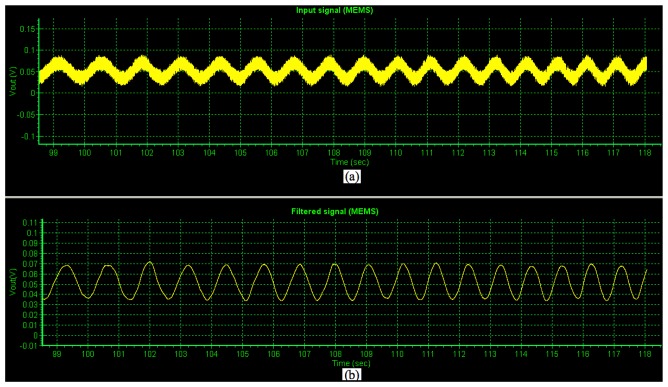
(**a**) Non-filtered and (**b**) filtered PCB output voltage (amplified 5,000 times) of the MEMS sensor generated by an alternating magnetic flux density with a maximum magnitude of 3,300 nT.

Later, a test of the non-filtered and filtered PCB output voltage signals (amplified 5,000 times) of the MEMS sensor generated by an alternating magnetic flux density (maximum magnitude of 3,300 nT) was obtained using the proposed virtual instrument. Figure 8a shows the non-filtered PCB output voltage (input) signal of the MEMS sensor, which contains components of electronic noise. On the other hand, Figure 8b depicts the filtered PCB output voltage signal of the MEMS sensor. This indicates the importance of our signal processing system and virtual instrument to decrease the noise electronic of the MEMS sensor.

## 3. Results and Discussion

This section presents the experimental set up to detect magnetic flux density during respiration in three intact rats anesthetized with ketamine and xylazine. For the experiments, we employed three male Sprague-Dawley rats (200–300 g) to obtain experimental measurements. Guidelines contained in the National Institutes of Health (NIH) *Guide for the Care and Use of Laboratory Animals* (NIH Publication No. 85–23, revised in 1985) and the “Norma Oficial Mexicana NOM-062-ZOO-1999”, were strictly followed. A mixture of ketamine (100 mg/kg, ip) and xylazine (5.2 mg/kg, ip) was applied to induce anesthesia. The level of anesthesia was verified throughout the whole experiment by testing for the lack of withdrawal reflexes and muscle tone. Doses of the ketamine-xylazine mixture were given when necessary. Body temperature was maintained at 36–37 °C.

We employed the improved MEMS sensor (with its virtual instrument) to detect the magnetic flux density during respiration in three intact rats anesthetized with ketamine. Rats were placed in a supine position and the MEMS sensor was placed about 4 to 6 mm from their thorax. The sensor was positioned at an angle of 45°. To avoid artifact movements the MEMS sensor did not touch the rat. The electromyogram (EMG) and the respiratory magnetogram were simultaneously recorded. The EMG was employed for the averaging of the respiratory magnetogram detected with the MEMS sensor.

**Figure 9.**
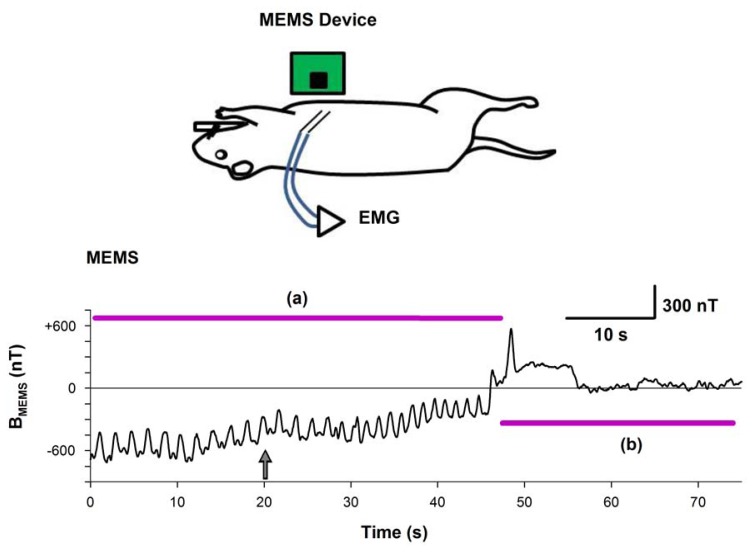

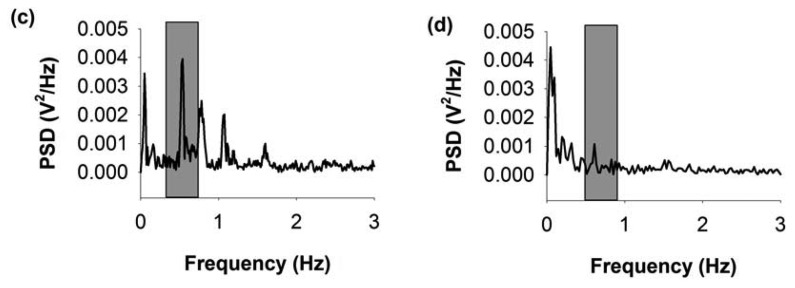
Upper panel, diagram of the experimental arrangement. (**a**) Magnetogram of a rat during respiration. (**b**) The same as (a) but after the rat was euthanized with an overdose of pentobarbitone. The computed power spectrum density (PSD) is depicted in (**c**) and (**d**) respectively. There are several frequency components during the respiration that subside after the euthanasia of the rat. We assumed the later condition as a “control” in which only background noise was present, while the respiration phase was considered as the noise+signal condition. The computed SNR between these signals was 17.62. B_MEMS_ indicates the magnetic flux density.

The upper panel of Figure 9 shows a diagram of the experimental arrangement. The MEMS sensor was positioned near to the intact thorax. The thoracic-muscle electromyogram (EMG) was simultaneously recorded and it served as a reference to compare both magnetic and electric signals generated during respiration. Figure 9a,b show the continuous respiratory magnetogram before and after the euthanasia of the rat. The processed signals have very good signal-to-noise ratio (SNR) to be detected online. We obtained a SNR = 17.6 for the recording depicted in Figure 9a relative to the background noise illustrated in Figure 9b when the rat was euthanized with an overdose of pentobarbital.

**Figure 10.**
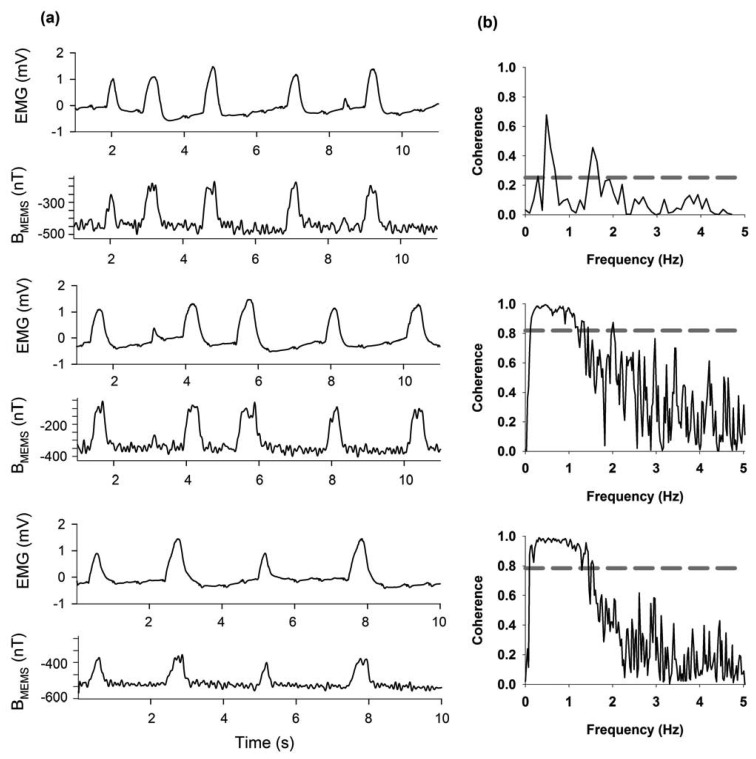
(**a**) Electromyogram recordings during intact respiration and the corresponding magnetograms detected with our MEMS device. The MEMS was placed 4 mm away from the thorax. Here we display the continuous recordings of three intact rats. (**b**) Coherence analysis for each pair of electromyogram (EMG)/MEMS recordings. The lower frequencies associated with the respiratory cycle exhibited the highest coherence between recordings. Interrupted lines represent the magnitude of a 95% confidence interval. B_MEMS_ indicates the magnetic flux density.

Figure 10a,b show continuous recordings of 10 s (bandwidth 0.3 Hz–10 kHz) for three different rats. Furthermore, we have included an analysis of the coherence between the electromyogram and the respiratory magnetogram, as shown in Figure 10b.

The response of the magnetic flux density obtained through of a respiratory magnetogram could help analyze the respiratory dynamics in different physiological aspects. Our semi-portable prototype could be used to find a respiratory magnetogram. Thus, this prototype could be employed by physiologist, physician, biologist and biomedical researchers.

Future research directions will include real-time non-invasive monitoring of magnetic flux density produced by the thoracic cage in anesthetized cats. In addition, reliability tests of the proposed prototype will be considered. For example, some tests will be necessary to examine whether the MEMS magnetic lead field is narrower than that of the EMG, as in the case of the magnetoencephalogram (MEG) lead field compared to the electroencephalogram (EEG) lead field, in which, the MEG “sees” an area on the cortex which is approximately 0.3 times that for the EEG [44], thus providing a superior spatial resolution for the MEG. If this were the case for our prototype, a possible biomedical application could be the selective detection of dysfunctional thoracic muscles in subjects with traumatic or dystrophic lesions affecting the respiration.

## 4. Conclusions

A semi-portable prototype for real-time non-invasive monitoring of rat respiratory magnetograms was presented. This prototype is made up of a MEMS sensor, a signal processing system implemented on a PCB, and a virtual instrument based on object-oriented programming. This programming offered advantages such as the integrity, compatibility, portability, and extendibility. It had capacity to filter small electromagnetic field signals, which avoided the use of a Faraday cage. It allowed the attenuation of magnetic noise through the use of different configurations of IIR filters, which were embedded in the software of the virtual instrument. The semi-portable prototype allowed the real-time detection of magnetic flux density close to 400 nanoteslas. With the designed system, the output signal of the MEMS sensor is easily conditioned. In addition, the rat respiratory magnetogram had a response behavior similar to that of its corresponding electromyogram (EMG). The use of our magnetic MEMS sensor offers several advantages over the EMG recording. The first is that it could be employed in non-invasive tests of magnetic flux density in biomedical applications that require not touch the skin of the subjects with EMG electrodes. The second is the size of the proposed prototype, which could be implemented for multi-site recordings on the proximity of the thorax. The third advantage is that our prototype is not expensive and it is easy to implement for custom applications.
